# Fetal Tetra-Amelia Birth: A Case Report

**DOI:** 10.1155/crog/7801322

**Published:** 2024-12-31

**Authors:** Eyob Asefa Belay, Anberbir Girma Asebot, Bezza Kedida Dabi

**Affiliations:** ^1^Department of Obstetrics and Gynecology, Nekemte Compressive Specialized Hospital, Nekemte, Ethiopia; ^2^Department of Obstetrics and Gynecology, Jimma University School of Medicine, Jimma, Ethiopia

**Keywords:** amelia, meromelia, Nekemte, teratogens

## Abstract

Fetal limb anomaly presentation varies greatly. It can present as amelia (complete absence of skeletal part of one or more limb), meromelia (partial absence of skeletal part of one or more limb), phocomelia (only rudimentary limb formed), and minor limb disorders like polydactyly. The complete absence of the four fetal limbs is extremely rare. Incidence of tetra-amelia is not well known, but it is mentioned to be 1–4 in 100,000 births in different literature. Etiopathogenesis of fetal tetra-amelia remains speculative. Tetra-amelia occurs either as tetra-amelia syndrome (when other organ systems are affected too) or isolated tetra-amelia. Tetra-amelia syndrome is more common than isolated tetra-amelia. It occurs secondary to genetic aberration or is sporadic. Genetic inheritance of tetra-amelia may present as autosomal dominance, autosomal recessive, or X-linked recessive. The protein coded on WTN 3 on chromosome 17q21 is important for fetal limb and other organ system formation. Mutation associated with the WTN 3 gene is a known cause for fetal limb malformation. Maternal diabetes, amniotic band syndrome, TORCH (toxoplasmosis, rubella cytomegalovirus, herpes simplex, and HIV) infection, alcohol consumption, and intrauterine exposure to some drugs like thalidomide, glucocorticoids, and sedatives are risk factors for limb malformation. Tetra-amelia can be diagnosed as early as the first trimester of pregnancy. Ultrasound imaging is a gold standard investigation to detect tetra-amelia. Tetra-amelia syndrome is associated with high mortality and morbidity than isolated tetra-amelia. In this case report, we present an extremely rare case, isolated tetra-amelia, born to G3P2 (Gravida 3, Para 2) mother at 36 weeks of gestation. It was diagnosed late in pregnancy at 34 weeks. The fetal organs (kidney, lung, abdominal wall, heart, vertebrae, and brain) were evaluated with ultrasound and were found healthy. She gave birth by cesarean section at 36 weeks of gestation for other obstetric indication. Isolated tetra-amelia is an extremely rare case. Early antenatal ultrasound fetal evaluation should be promoted. Early detection of tetra-amelia helps to provide parental counselling and option of management timely. Parental counselling should involve how to care for the neonate with tetra-amelia. Community awareness creation is important to decrease social stigma against babies with tetra-amelia. In such rare cases, it is important to alert public health researchers for possible further epidemiological study.

## 1. Introduction

Fetal limb anomaly presentation varies greatly. It can present as amelia (complete absence of skeletal part of one or more limb), meromelia (partial absence of skeletal part of one or more limb), phocomelia (intermediate part of limb with the hand or foot directly attached to the trunk), and minor limb disorders like polydactyly [1–4]. Fetal amelia is a congenital anomaly defined as the complete absence of a skeletal part of limb [1]. It occurs when there is either prevented or arrested fetal limb formation in the early embryogenesis period, 24 to 36 days post fertilization [5]. Commonly, a single fetal limb is affected. The complete absence of the four fetal limbs is extremely rare [1, 6]. The incidence of amelia is not well known but commonly mentioned to be 0.05 to 0.095 in 10,000 live births [1, 2]. Tetra-amelia is the rarest form of fetal amelia with incidence of 1–4 in 100,000 births [7]. Fetal tetra-amelia is often associated with multiple organ system anomaly and presents as fetal tetra-amelia syndrome. The face, brain, vertebrae, kidney, heart, and abdominal wall are commonly involved organs [1, 2]. Rarely, it is seen as isolated tetra-amelia [7]. Etiopathogenesis of fetal tetra-amelia is not well known but believed to be multifactorial. The genetic aspect and mode of inheritance remain speculative [8]. Proteins coded by the WTN 3 gene on chromosome 17q21 is important for fetal limbs and other organ system development during embryogenesis [9]. Gene mutation involving WTN 3 will result in disruption of fetal limbs and other organ system development [7, 10, 11]. Other genetic inheritance involving autosomal recessive, autosomal dominant, and X-linked recessive is also associated with fetal tetra-amelia [2, 7]. Teratogens linked to fetal amelia are not fully studied yet. Maternal diabetics, amniotic band syndrome, TORCH (toxoplasmosis, rubella cytomegalovirus, herpes simplex, and HIV) infection, abnormal genetic test history, harmful chemical exposure, harmful drug intake (e.g., thalidomide, sedative, and glucocorticoids), and alcohol consumption are risk factors for fetal limb anomaly [1, 6]. In the 1960s, studies have indicated that thalidomide was a known risk factor for phocomelia [12]. Currently, thalidomide is out of the market. Early diagnosis of tetra-amelia is an important milestone for management option. Ultrasound imaging, preferably combined transvaginal and transabdominal approach, is a gold standard for early tetra-amelia diagnosis [6]. Such an extremely rare case has prompted us to present this case report. In addition to its rarity, newborn with tetra-amelia congenital anomaly needs future physical and psychological care from family and community.

## 2. Case Presentation

This is a case presentation of a G3P2 (Gravida 3, Para 2) mother who presented to a prenatal care clinic at the Nekemte Compressive Specialized Hospital at 8 months of amenorrhea. She has antenatal follow-up during the index pregnancy, which she started late in pregnancy at gestational age 34 weeks. On the day of antenatal follow-up, she was evaluated with ultrasound. Ultrasound finding was singleton intrauterine fetus, placenta was fundal, head occupies in the fundus, and biparietal and abdominal circumference was 35 and 34 weeks, respectively. There were upper and lower extremities; fetal brain, spine, kidney, liver, and lung look normal. There was no visible abdominal wall defect. Fetal biophysical profile was reassuring. All basic investigations were normal. On CBC, hemoglobin = 14.5 g/dL and platelet (PLT) = 225,000. Random blood sugar (RBS) was 110 mg/dL, and fasting blood sugar (FBS) was 93 mg/dL. Blood group was AB positive. HIV test, HBsAg, and VDRL were negative. Urine analysis finding was normal. Her weight was 56 kg. With these evidences, fetal tetra-amelia in the third trimester was diagnosed. She was counselled on the outcome, and she decided to continue pregnancy. There were no pregnancy-associated danger signs. She had one previous cesarean delivery for the second baby, which was indicated for fetal malpresentation. She has no chronic medical illness and history of drug allergy history. She has no family history of congenital anomalies. She has two healthy babies, a female and a boy. She works in a government office.

**Figure 1:**
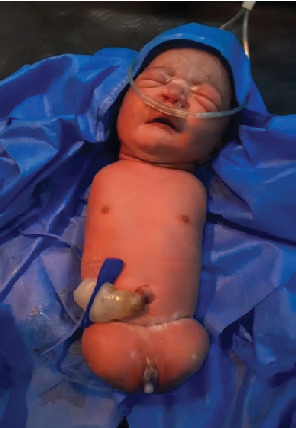
The neonate's four limbs are missing.

Two weeks later, she presented to the labor and delivery unit with chief complaint of pushing down pain of 3 h duration and passage of liquor of 1 h duration. Upon evaluation, all vital signs were stable. BP = 120/70 mmHg, pulse rate (PR) = 96 bpm, respiration rate (RR) = 22 bpm, and temperature (*T*°) = 36.5°C. On HEENT (head, eyes, ears, nose, and throat) exam, conjunctiva was pink and anicteric sclera. On abdominal examination, gravid uterus was 36 weeks sized, fetal head is palpable in the uterine fundus, fetal back on the left side of the mother, and fetal heartbeat (FHB) was 120–124 bpm. There was significant suprapubic tenderness along an old transverse surgical scar. On the genitourinary system, cervix was 3 cm dilated, blood stained the amniotic fluid, and station was at −1. Uterine dehiscence in the latent first stage of labor was diagnosed. After she was resuscitated with crystalline fluid, she was taken to the operating room for cesarean section. Intraoperatively, there was about 5-cm uterine dehiscence on the left side old scar. Incision was made on the right side of the lower uterine segment, and the neonate was delivered. The uterus was repaired along with dehiscence part. A female neonate with all four limbs missing, otherwise healthy, was born. Fetal weight was 2 kg. APGAR (appearance, pulse, grimace, activity, and respiration) score was 9 and 10 at the first and fifth minute, respectively. The neonate was taken to the neonatal ward for further evaluation. A pediatrician evaluated, and there were no additional problems diagnosed. The mother and baby were discharged home after 5 days of stay in the hospital. The mother and neonate were evaluated 1 week postnatal period, and both the mother and baby were found fine. Images of the neonate were taken immediately after cesarean section.  Figure 1 shows the neonate's four limbs are missing.

## 3. Patient Perspectives

“I was 8 month pregnant when I visited a doctor at Nekemte compressive specialized hospital. I was examined with ultrasound, blood and urine tests. My doctor take extended time for ultrasound examination than I expected. After a while the doctor informed me result of ultrasound imaging result. I was shocked that doctor informed the fetus has no legs and upper arms. I was with my husband and took more time to discuss with the doctor. The doctor told us that the fetus has no other associated anomaly than legs and upper arms. It was late pregnancy to consider termination. After discussion, we agreed to continue ante-natal care follow up and to be ready for neonatal care. At 36 weeks of gestation, I experienced pushing down pain. I was admitted to labor and delivery unit for emergency cesarean delivery. I gave birth to an infant with all extremity missing. Otherwise, the infant was active and started breast feeding. Initially, it was difficult to accept. Hours later, I and my husband accepted that it is a gift of god. I prayed that let god strengthen. I was discharged home from hospital days later. I appreciate my doctors for everything they did for me and my baby. Let God bless them!”

## 4. Discussion

This case report presents an extremely rare case of skeletal congenital anomaly, a fetal tetra-amelia. Amelia is defined as the complete absence of skeletal part of a limb or limbs, and meromelia is when there is the absence of some part of limbs' skeleton [1, 2, 13]. Amelia is heterogeneous in its occurrence. It is either sporadic or an inheritance of genetics [13]. Autosomal recessive, autosomal dominant, and X-linked recessive inheritance are mentioned as the genetic heterogeneity of conditions [1, 2, 9]. Genetic inheritance is associated with multiple organ anomalies [11]. Prenatal thalidomide exposure, alcohol consumption during pregnancy, smoking, diabetic mellitus, TORCH infection, and amniotic band syndrome are also causes of fetal limb abnormalities [5, 6, 9, 13]. Karyotyping and maternal serum analytes are important as additional investigation in tetra-amelia syndromes [6, 7, 14]. In this case, there was no prenatal exposure to alcohol and thalidomide. Also, the mother is not a diabetic patient. There are no genetic tests in our setup. Genetic aberration is associated with multiple abnormalities and recurrences. So, it is difficult to suggest future case recurrence and to ascertain whether genetics is the cause of index case. There are no TORCH infection tests in our health facility, and her status is unknown.

Fetal amelia is commonly associated with other congenital anomalies. The brain, vertebrae, heart, kidney, and face are commonly affected organs [7, 13]. If one or more of these organs are affected, fetal outcome will be worse. Ultrasonography imaging is a gold standard to detect limb anomaly [15]. Tetra-amelia can be detected as early as the first trimester [7]. Once tetra-amelia is detected, it is mandatory to have anatomic scanning of other organ systems because tetra-amelia commonly presents as a syndrome. The brain, vertebrae, heart, kidney, face, lung, and abdominal wall are affected when tetra-amelia syndrome is the cause. Maternal serum alpha-fetoprotein, human chorionic glycoprotein, and unconjugated estriol are auxiliary tests which suggest possibility of other associated chromosomal aberration and congenital anomalies like neural tube defect and anterior abdominal wall defect [16]. Additional imaging (CT scan and MRI) and investigation may be needed to characterize other affected organs. Fetal tetra-amelia syndrome is associated with high mortality and morbidity, and termination of pregnancy is a better option of management. If isolated fetal tetra-amelia is considered, parental counselling, whether to terminate or continue pregnancy, should be provided. In our case, fetal tetra-amelia was detected in the third trimester of pregnancy, at 34 weeks of gestation, by ultrasound imaging. Upon evaluation, fetal brain, vertebrae, heart, kidney, face, lung, and abdominal wall were normal. So, isolated tetra-amelia was considered which is a far rare case. After delivery, the neonate was evaluated by a pediatrician and no additional anomaly detected. A tetra-amelia neonate, otherwise healthy, which was consistent with antenatal diagnosis was born. With this finding, the neonate was diagnosed with isolated fetal tetra-amelia.

## 5. Conclusion

Isolated tetra-amelia is an extremely rare case. Early antenatal ultrasound fetal evaluation should be promoted. Early detection of tetra-amelia helps to provide parental counselling and option of management timely. Parental counselling should involve how to care for the neonate with tetra-amelia. Community awareness creation is important to decrease social stigma against babies with tetra-amelia. In such rare cases, it is important to alert public health researchers for possible further epidemiological study.

## Data Availability

Data used during the current study are available from the corresponding author upon reasonable request.
